# Cancer and lymphatic marker FOXC2 drives wound healing and fibrotic tissue formation

**DOI:** 10.3389/fphys.2024.1427113

**Published:** 2024-10-15

**Authors:** Maia B. Granoski, Katharina S. Fischer, William W. Hahn, Dharshan Sivaraj, Hudson C. Kussie, Filiberto Quintero, Abdelrahman M. Alsharif, Eamonn McKenna, Jonathan P. Yasmeh, Andrew C. Hostler, Maria Gracia Mora Pinos, Robert P. Erickson, Marlys H. Witte, Kellen Chen, Geoffrey C. Gurtner

**Affiliations:** Department of Surgery, University of Arizona, Tucson, AZ, United States

**Keywords:** FOXC2, lymphatics, wound healing, inflammation, fibrosis

## Abstract

**Introduction:**

The FOXC2 transcription factor has been tied to a wide range of disease states, serving as a promising prognostic biomarker associated with aggressive basal-like human breast cancers (increased cancer invasion and metastasis). Dysregulation of FOXC2 expression has also been found to promote defects in lymphatic remodeling and hyperplastic lymphedema-distichiasis (LD). Since chronic lymphedema is a forerunner of several malignancies and cancers have been known to arise from poorly healing chronic wounds (e.g., Marjolin ulcers), we examined the effect of Foxc2 dysfunction on skin wound healing.

**Methods:**

We used our splinted excisional wounding model that mimics human-like wound healing on wildtype and Foxc2^+/−^ mice (n = 4), which demonstrate incomplete lymphatic vasculature and lymphatic dysfunction. Wound size was measured over the course of 18 days. Tissue was explanted from both groups at post-operative day (POD) 14 and 18 and stained with Masson’s Trichrome to assess scar formation, Picrosirius Red for dermal integrity, or immunofluorescence to assess lymphatic (LYVE1) cell populations.

**Results:**

Wildtype mice completely healed by POD 14, while Foxc2^+/−^mice did not completely heal until POD18. Scar area of healed Foxc2^+/−^mice (POD 18) was larger than that of healed wild-type mice (POD 14; *p* = 0.0294). At POD 14, collagen "bers in the scars of Foxc2^+/−^mice to be narrower (*p* = 0.0117) and more highly aligned (*p* = 0.0110), indicating signi"cantly more "brosis in these mice. Collagen "bers in both groups became longer (*p* = 0.0116) and wider (*p* = 0.0020) from POD 14 to 18, indicating a temporal evolution of "brosis. Foxc2^+/−^mice also had lower numbers of LYVE1+, F4/80+ and CD4+ cells compared to wildtype mice.

**Discussion:**

Individuals over 65 years old are more likely to develop cancer and are highly susceptible to developing chronic wounds. Here, we found that FOXC2, which is tied to cancer metastasis and lymphatic dysregulation, also impairs wound healing and promotes "brotic tissue architecture. With FOXC2 proposed as a potential therapeutic target for cancer metastasis, its downstream systemic effects should be considered against the increased chance of developing nonhealing wounds. Further delineation of the microenvironment, cellular events, and molecular signals during normal and Foxc2-associated abnormal wound healing will improve clinical therapies targeting this important marker.

## Introduction

Wound repair is a complex and highly coordinated biological process that engages many different physiological systems during interdependent phases of inflammation, tissue formation, and tissue remodeling (Chen et al., 2022). Lymphatic dysfunction can result in lymphedema, characterized by chronic and progressive accumulation of lymph in the body which causes swelling in the limbs, and it has been speculated to be linked to increased wound development due to a decreased capacity to deliver or remove nutrients in affected tissue (Aldrich et al., 2020; Olszewski et al., 2012). Although edema is a part of wound healing in early stages, chronic edema can impair blood flow to the wound site and prevent proper drainage of the inflamed area (Jiang et al., 2018). Lymphedemic conditions can result in the development of chronic wounds, epithelial erosion, and amputation of affected limbs (Grada and Phillips, 2017). Chronic wounds do not progress through the normal healing stages, and instead enter a state of unresolved healing and continuous inflammation (Gurtner et al., 2008). Understanding the role that the lymphatic system plays in the progression of the wound-healing process is increasingly relevant to the treatment and prevention of chronic wound development.

Forkhead-Box C2 (*FOXC2*) is a member of the forkhead-box transcription factor family that plays a fundamental role in the development of many tissues during embryogenesis, including adipose tissue, the nervous system, and the lymphatic system (Tavian et al., 2020). Systemic dysregulation of *FOXC2* has been found to promote defects in lymphatic vasculature and hereditary lymphedema-distichiasis syndrome, which presents as distichiasis (double rows of eyelashes) and lower-limb lymphedema that develops in adolescence (Tavian et al., 2016; Fang et al., 2000; Erickson et al., 2001). *FOXC2* has also recently emerged as a potential biomarker linked to the progression of several cancers and is associated with tumor cell proliferation, drug resistance, and initiation of the epithelial-mesenchymal transition (EMT) that takes place before cancer cell invasion and metastasis (Hargadon and Strong, 2023; Mani et al., 2007; Zhang et al., 2022). Murine heterozygous *Foxc2* genetic knockdowns have been previously been used as a model for human lymphedema-distichiasis and demonstrate similar phenotypic symptoms, including hyperplasia of lymphatics and lymph nodes (Kriederman et al., 2003). Homozygous knockout animals die embryologically or shortly after birth due to complications from skeletal abnormalities (Kriederman et al., 2003). Previous studies have interrogated the relationship between lymphatic vasculature and VEGF or immune signaling in the context of diabetic and corneal wound healing (Brunner et al., 2023; Güç et al., 2017; Maruyama et al., 2007; Maruyama et al., 2005). Others have investigated treatments that either support or hinder lymphatic vasculature infiltration during healing However, the downstream side effects of FOXC2 inhibition in contexts such as wound healing have not yet been investigated.

Here, using an established murine model of excisional wounding, we show that mice with targeted inactivation of the *Foxc2* gene have poorer healing capabilities and lower scar tissue quality compared to wildtype mice as measured by time to wound closure, collagen architecture, and inflammatory cell infiltration. These findings imply that the lymphatic system contributes significantly to wound healing and that lymphatic impairment can lead to complications in healing and the potential development of chronic wounds.

## Materials and methods

### Animals

Genetically lymphatic-deficient mice (C57BL/6J-*Foxc2* ^+/−^) were obtained from Jackson Laboratories (Bar Harbor, ME). These heterozygous mice possess a genetic deletion of one copy of the *Foxc2* gene and produce very low levels of *Foxc2*, representing a model of lymphatic deficiency similar to lymphedema-distichiasis (LD) and impaired lymphatic vasculature. Previous studies have found that homozygous deletion of *Foxc2* is lethal, and homozygous null animals die either embryologically or shortly after birth (Iida et al., 1997). Heterozygous animals are viable and have been phenotypically characterized as having lymphatic impairments (Kriederman et al., 2003). Animal care was provided according to the University of Arizona College of Medicine guidelines for use of laboratory animals with Institutional Animal Care and Use Committee (IACUC) protocol 2021-0828.

### Phenotype

Staining with Evans blue dye (EBD) was performed to confirm lymphatic abnormalities in heterozygote (*Foxc2* ^+/−^) mice. EBD is a vital dye that binds to tissue proteins and is selectively absorbed into the initial lymphatic vessels. EBD was injected into all four paws, the ears, and the snout serially to visualize the regional lymphatic systems and central collection system. The peripheral lymphatic vasculature was examined by dissection of the popliteal, axillary, and jugular areas (Kriederman et al., 2003) (Supplementary Figure S1).

### *In Vivo* stented excisional wound model

We utilized 15-week-old C57BL/6-FOXC2 (*Foxc2* ^+/−^) mice in an excisional wounding model that closely mimics human wound healing. Splinted full thickness wounds were created as described by Fischer et al. (2023). A sterile 6 mm biopsy punch tool was used to create a full thickness wound on each side of the dorsal midline. Each wound was splinted using a circular silicone ring cut from a 0.5 mm silicone sheet. Rings had an outer diameter of 15 mm and an inner diameter of 10 mm. Splints were centered around the wound, attached using surgical glue, and then secured with 8 sutures to prevent wound contracture. Wounds were covered with sterile occlusive dressing (Tegaderm, 3M, St. Paul, MN, United States of America). Four *Foxc2* ^+/−^ mice and four wildtype (C57BL/6) control mice were used for each of the two experimental time points, and two dorsal excisional wounds were created per mouse, resulting in a total of 8 wounds in the *Foxc2* ^+/−^ group and 8 wounds in the control group at each time point. Two experimental endpoints, post-operative day (POD) 14 and POD18, were chosen based on the observed healing patterns of wildtype and *Foxc2* ^+/−^ mice. A total of 16 mice and 32 wounds were used for analysis. Digital photographs were taken of the wound on POD0 and repeated every other day until wound closure. Wound areas were quantified using Fiji and expressed as a percentage of the original area. Wound healing curves were generated for each experimental group by graphing wound size percentage vs. time.

### Histological analysis of collagen content and architecture

Wound area tissue was harvested at POD14 and POD18. After harvesting, samples were placed in cassettes, fixed in 4% paraformaldehyde, dehydrated and then embedded in Optimal Temperature Cutting (OCT) compound (TissueTek, Sakura Finetek, Torrance, CA) for frozen sectioning. Hematoxylin and Eosin (H&E) and Masson’s Trichrome staining were performed according to the manufacturer’s recommendations. Images were taken with a LeicaDMI6000 upright microscope using Brightfield microscopy. Some samples were excluded from histological analysis due to poor tissue quality. The average depth of scar tissue formation was analyzed using Fiji to measure the collagen area of the Trichrome images and divide this measurement by the scar length. We implemented an algorithm in MATLAB to deconvolve the color information of each trichrome image. This algorithm allows for the determination of a color matrix based on the stain-specific RGB light absorption of the samples (Ruifrok and Johnston, 2001). We also performed Picrosirius Red (Sigma Aldrich) staining, and images were captured with a LeicaDMI6000 upright microscope using polarized light microscopy. Polarized light was oriented to maximally display fibers parallel to the skin surface. Collagen fiber quantification was performed using CT-FIRE and CurveAlign, an open-source software package for automatic segmentation and quantification of individual collagen fibers (Liu et al., 2017). CurveAlign quantifies collagen fiber angles and the strength of alignment within an image, while CT-Fire is used to analyze individual fiber metrics such as length, width, angle and curvature. The average fiber parameters for each mouse were used for statistical analysis.

### Immunohistochemistry

Immunofluorescent staining was performed using primary antibodies F4/80 (1:100 dilution, AbCam, Ab6640), LYVE1 (1:100 dilution, Thermo Fisher Scientific, #14-0443-82), CD4 (1:100 dilution, Thermo Fisher Scientific, #PA5-87425), CD8 (1:100 dilution, Thermo Fisher Scientific, #14-0081-82) and CD19 (1:100 dilution, Thermo Fisher Scientific, #MA5-29094). The percentage of fluorescent area was quantified using a custom MATLAB image processing code written by the authors and previously published (Chen et al., 2018). All immunofluorescent images shown are representative images.

### Statistical analysis

Statistical analysis was performed in Prism10 (GraphPad, San Diego, California) using either an Unpaired Student’s t-test or two-way analysis of variance (ANOVA). Data are presented as means ± SEM. Values of *p < 0.05 were considered statistically significant.

## Results

### Foxc2^+/−^ mice heal more slowly than wildtype mice

**FIGURE 1:**
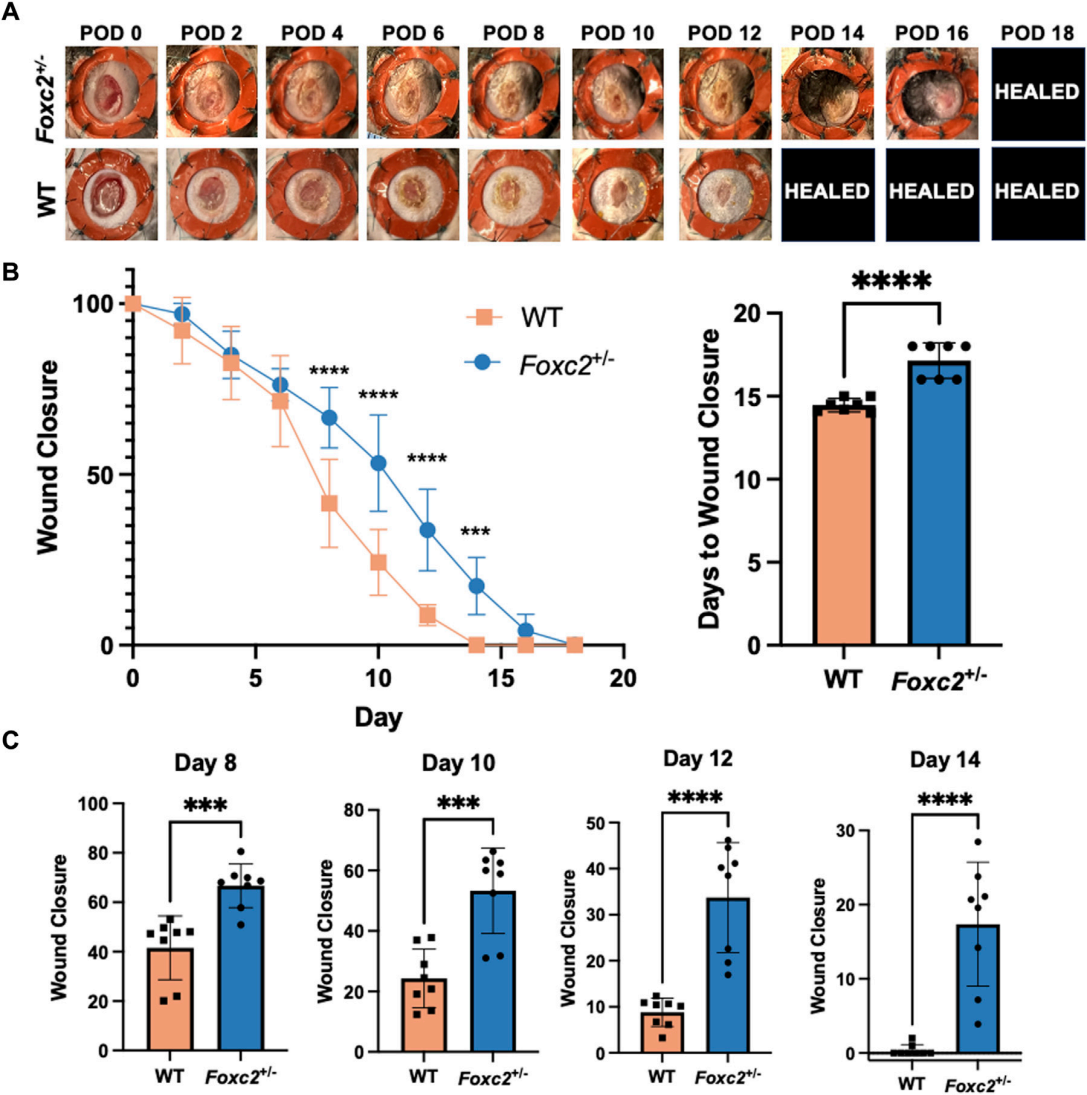
**(A)** Representative images of the wounds over time by group. POD, postoperative day. Healed = healed wound that has closed. **(B)** Quantification of wound area over time by group. **(C)** Wound area size at postoperative (POD) 8, 10, 12 and 14.

To assess wound healing of *Foxc2* ^+/−^ mice, we measured wound area change over time by analyzing digital photographs taken during each dressing change (Figure 1A). The wound size was represented as an average size of 8 wounds per group (4 mice per group, 2 wounds per mouse). Wound areas of *Foxc2* ^+/−^ mice were significantly larger than those of wildtype mice at PODs 8 (*p* < 0.0001), 10 (*p* < 0.0001), 12 (*p* < 0.0001), and 14 (*p* = 0.0002), indicating delayed wound healing across various time points (Figure 1B). The wound size percentage at PODs 8, 10, 12 and 14 are also shown as individual bar graphs to further demonstrate this significant difference (Figure 1C). The mean time for complete wound healing of *Foxc2* ^+/−^ mice was 18 ± 0.5 days, which was significantly longer than 14 ± 0.7 days for wildtype mice (*p* = 0.0104) (Figure 1B).

### Foxc2^+/−^ mice have poor lymphatic vessel infiltration and fewer inflammatory cells at the wound site than wildtype mice

**FIGURE 2:**
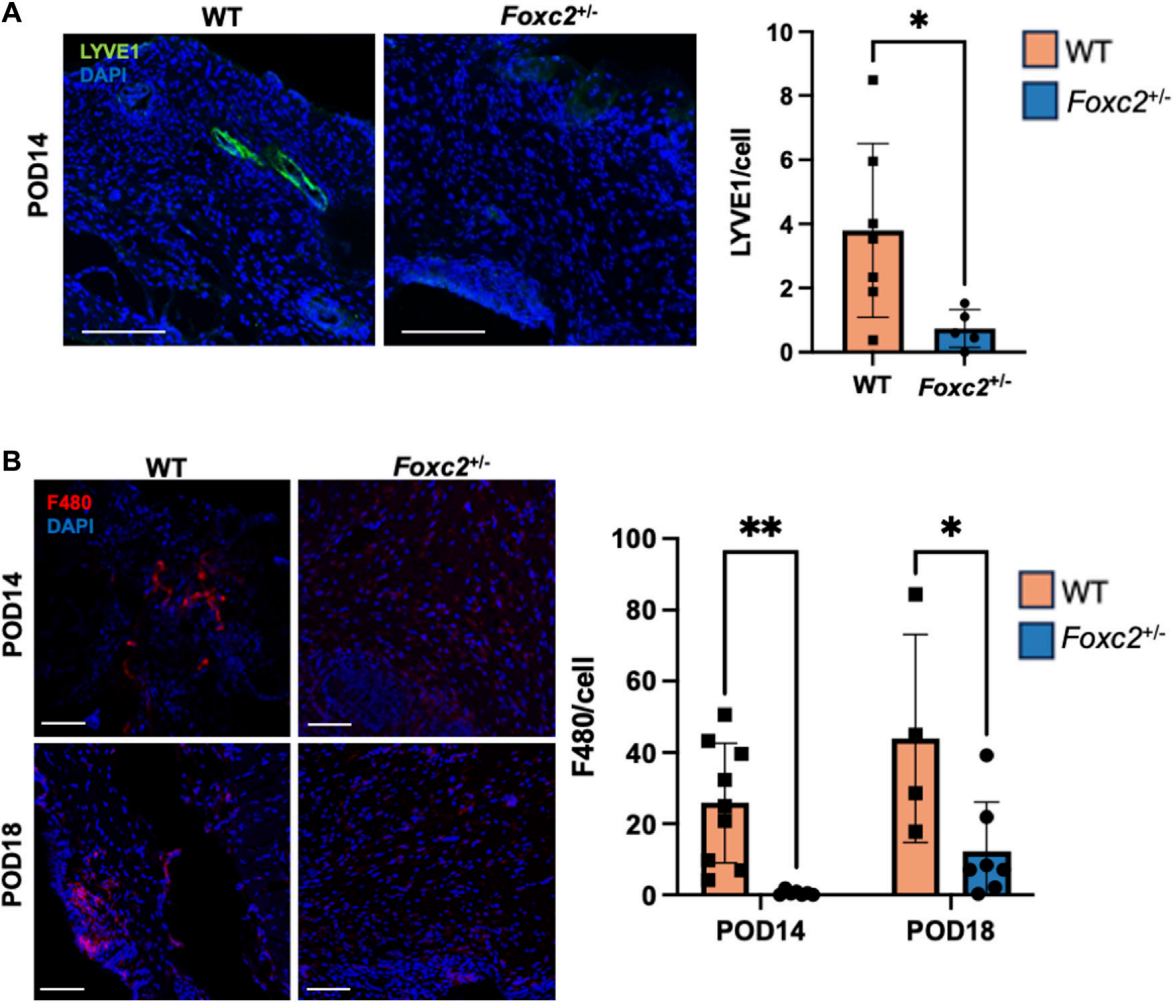
**(A)** Immunostaining for LYVE1 in tissue sections on POD14. **(B)** Immunostaining for F480 in tissue sections on POD14 and 18. Scale bars 50µm. Quantification of percent area positive for marker in each section. Data are presented as ± SEM, *p < 0.05.

**FIGURE 3:**
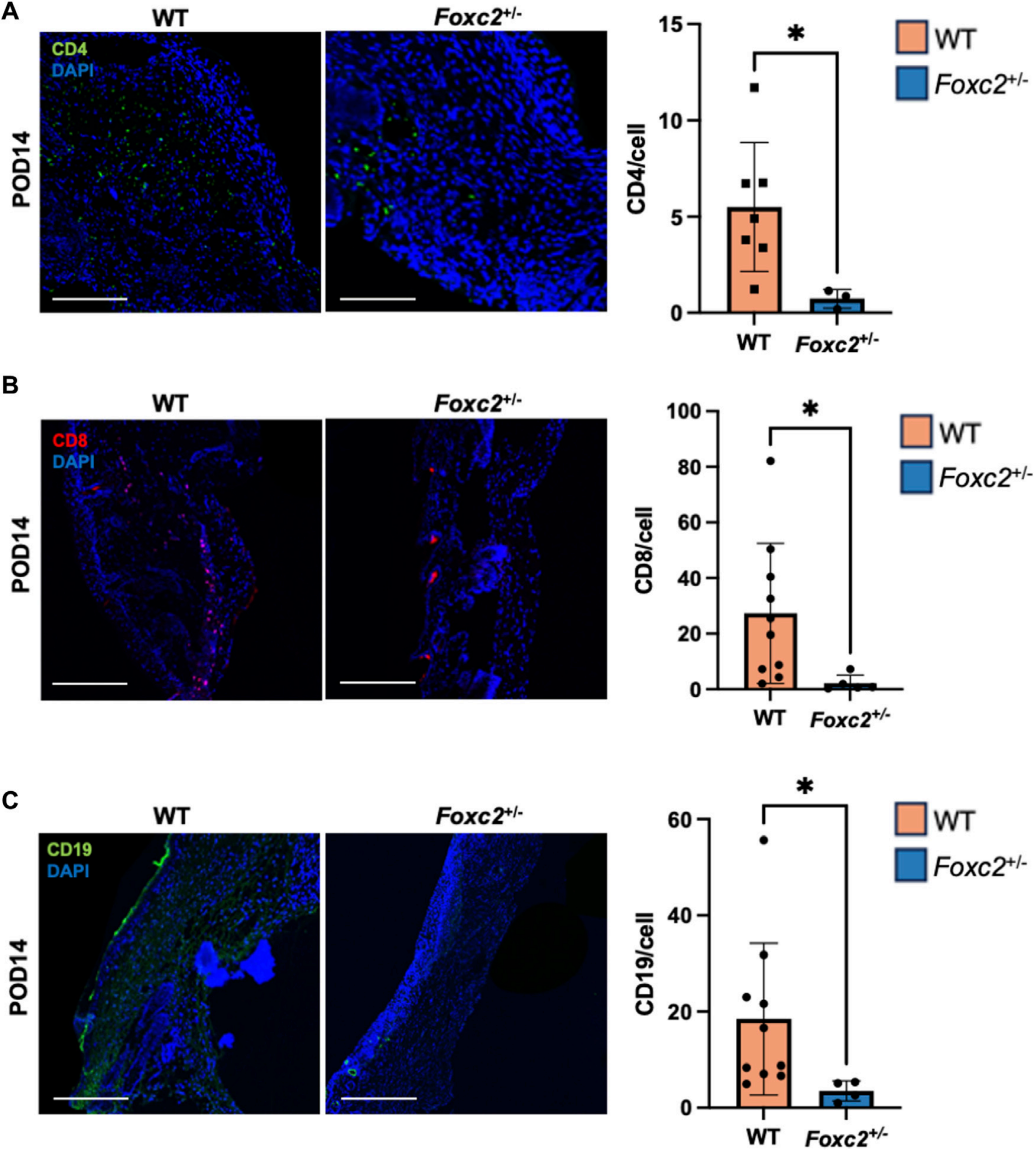
**(A)** Immunostaining for CD4, **(B)** CD8 and **(C)** CD19 in tissue sections on POD14. Scale bars are 50µm. Quantification of percent area positive for marker in each section. Data are presented as as ± SEM, *p < 0.05.

To assess the factors promoting wound healing in *Foxc2* ^+/−^ mice, we performed immunostaining of LYVE1, a well-established lymphatic receptor; CD4, a surface marker on immune cells such as T-helper cells, NK cells, monocytes and innate lymphoid cells; CD8, a surface marker on cytotoxic T-cells; CD19, a marker on B-cells; and F4/80, a common murine macrophage marker (Dos Anjos Cassado, 2017; Jackson, 2004; Künzli and Masopust, 2023). At POD14, levels of LYVE1+ cells (lymphatic vasculature) were significantly lower in *Foxc2* ^+/−^ mice (*p* = 0.0342, Figure 2A), indicating impaired lymphatic infiltration at the wound site. At both PODs 14 and 18, levels of F4/80+ cells (macrophages) were significantly reduced in *Foxc2* ^+/−^ mice compared to WT (*p* = 0.0322, *p* = 0.0014; Figure 2B). At POD14, CD4^+^ cells (*p* = 0.0452, Figure 3A), CD8^+^ cells (*p* = 0.0479, Figure 3B), and CD19^+^ cells (*p* = 0.0154, Figure 3C) were also significantly reduced in *Foxc2* ^+/−^ mice, indicating an impaired adaptive immune response during the healing process compared to wildtype mice. Together, these findings confirm lymphatic dysregulation and suggest that disruption of lymphatic architecture distinctly affected macrophage and immune cell recruitment within the wounds.

### Collagen deposition increases over time in both Foxc2^+/−^ mice and wildtype mice

**FIGURE 4:**
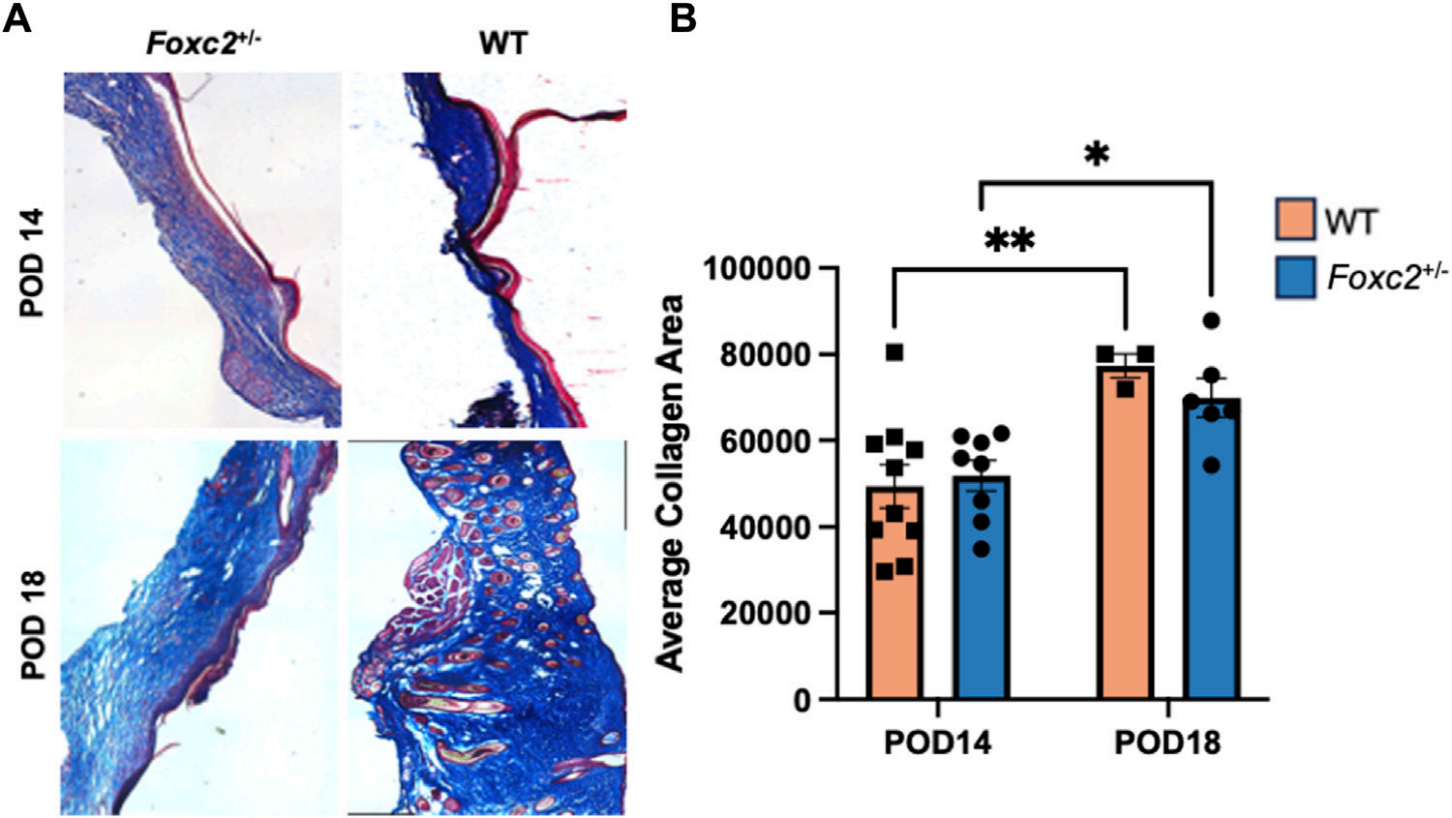
**(A)** Masson’s trichrome staining of representative tissue sections showing dermal structure of wildtype and *Foxc2* ^+/−^ wounds at POD14 and 18. **(B)** Analysis for total area positive for collagen at POD14 and 18.

We analyzed the dermal structure of murine scar tissue from Masson’s Trichrome staining and imaging (Figure 4A). Histological collagen area of *Foxc2* ^+/−^ mice was not significantly different from that of WT mice at either POD14 or POD18 (Figure 4B). However, the trichrome collagen areas of both *Foxc2* ^+/−^ and WT mice increased significantly over time (from POD14 to POD18) (*p* = 0.0206; *p* = 0.0098) (Figure 4B). These results demonstrated that collagen deposition increases at the wound site over time in both groups.

### Foxc2^+/−^ mice have narrower and more highly aligned collagen fibers than wildtype mice during healing

**FIGURE 5:**
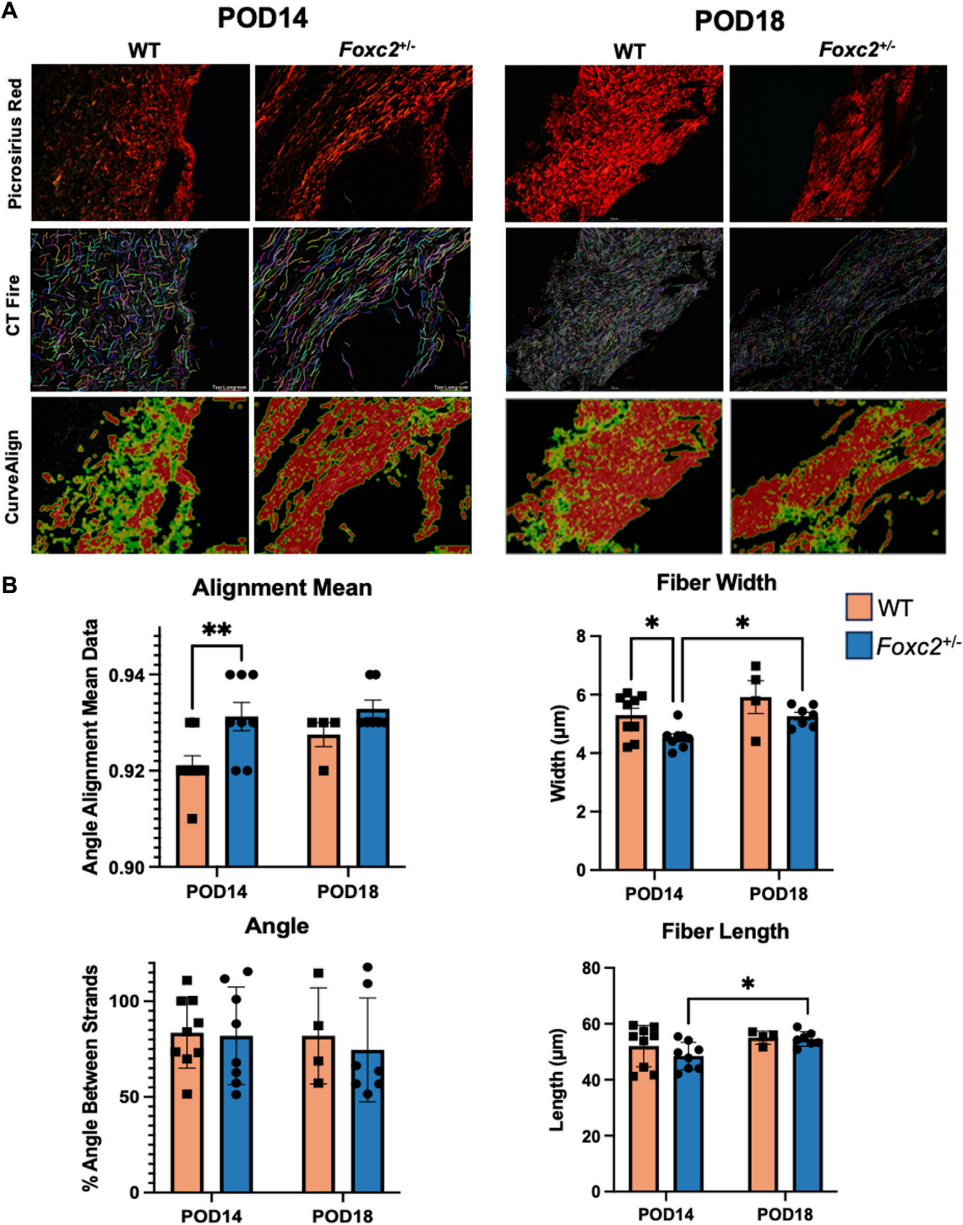
**(A)** Picrosirius red staining and comparison of wildtype and *Foxc2* ^*+/−*^ wounds at POD14 and 18 using collagen algorithms CTFire and CurveAlign. **(B)** Quantification of fiber alignment, width, angle skewness, and length.

We evaluated POD14 and POD18 wounds using picrosirius red staining to measure the collagen density and orientation of the scar collagen tissue architecture (Figure 5A). Using CT-Fire, we determined that collagen fibers were significantly narrower (*p* = 0.0174) and more highly aligned (*p* = 0.0110) in the scars of *Foxc2* ^+/−^ mice at POD14 compared to WT (Figure 5B). *Foxc2* ^+/−^ mice also demonstrated longer and wider collagen fibers at POD18 compared to POD14 (*p* = 0.0352) (Figure 5B). No significant differences in fiber length, width, angle or alignment were found between *Foxc2* ^+/−^ and wildtype mice at POD18 (Figure 5B). Because collagen fibers in unwounded skin are randomly aligned in a “basket-weave” orientation (Barrera et al., 2021; Corr and Hart, 2013), the significantly more aligned collagen network of the wounds in *Foxc2* ^+/−^ mice indicated a poorer quality of tissue and suggested an impaired healing capacity compared to wildtype mice.

## Discussion

While the role of blood vessel angiogenesis in wound healing has been well studied (DiPietro, 2016; Shi et al., 2023), the impact of lymphatic angiogenesis on the wound healing process has been largely uncharacterized. As a result, little is known about the molecular events that drive lymphatic processes known to occur during normal wound healing, such as lymphangiogenesis. While studies have previously characterized the morphology of lymph regeneration during normal wound healing and investigated cytokine signaling in a *k-cyclin* ^*+/−*^ lymphatic deficient model (Nogami et al., 2009; Komatsu et al., 2017; Kimura et al., 2013), the involvement of the transcription factor Foxc2 in the wound healing process has yet to be studied. Here, we found that lymphatic disruption in a murine *Foxc2* knockdown model caused a significant decrease in wound healing capabilities and immune cell recruitment that persisted to late time points. Although inflammatory cells are canonically thought to be important during the initial, early phase of wound healing, we surprisingly found that *Foxc2* ^+/−^ and WT mice healed similarly early, and healing rates did not diverge until POD8 to POD14. At POD 14, we observed persisting significant differences in the number of macrophages, CD4^+^ cells, CD8^+^ cells and CD19^+^ cells. CD19^+^ B-cells specifically have been found to support wound healing by producing cytokines, such as IL-10, that modulate the immune response during the healing process (Debes and McGettigan, 2019), and CD8^+^ T-cells maintain localized protection from wound infection during the process of repair (Osborn et al., 2019). Our findings also agree with Kimura et al., who found levels of IL-10 to be significantly reduced in a *k-cyclin* knockdown model of lymphatically deficient mice (Kimura et al., 2013). These findings suggest that targeting the lymphatic system affects immune cell activity which then leads to impaired late stage wound healing. Although immune cells are typically understood to be recruited through the blood vasculature and bloodstream, our findings demonstrate that macrophage, T cell, and B cell recruitment and delivery may also occur via the lymphatic vasculature and lymph stream.

Macrophages promote both angiogenesis and lymphangiogensis in wound healing by secreting factors (e.g., VEGF and TGFB-1) as well as recruiting CD4^+^ cells, CD8^+^ cells, and other lymphocytes to the wound site (Corliss et al., 2016; Fujiwara and Kobayashi, 2005; Krzyszczyk et al., 2018; Duong et al., 2021). Our findings agree with most previous studies that have observed lower macrophage counts associated with defects in lymphatic remodeling, decreased lymphangiogenesis, and impaired wound healing capabilities in a diabetic model (Maruyama et al., 2007; Ran and Montgomery, 2012). Macrophages have also been shown to support lymphangiogenesis in the context of corneal wound healing.

While lymphocytes have not previously been known to be vital to wound closure or scar formation, lymphatic growth at the wound site is necessary for the transport of lymphocytes to the wounded tissue (Maruyama et al., 2005; Schäffer and Barbul, 1998), and their presence at the wound site is critical to the prevention of infection and the maintenance of the inflammatory state during healing (Schäffer and Barbul, 1998; Chen et al., 2014). A breakdown in lymphangiogenesis at the wound site likely leads to a failure in lymphocyte transport to the affected tissue, causing lower overall levels of inflammation at the site. These findings imply that the impaired wound healing capabilities we observed here are likely due to a similar breakdown of immune and lymphatic signaling. Interestingly, lymphedema in humans is characterized by a prolonged inflammatory response and has been shown to result in a significant increase in immune cell count in the affected tissue (Jiang et al., 2018; Ly et al., 2019). FOXC2 impairment has similarly been associated with increased macrophage and T cell infiltration in the context of experimental colitis but decreased systemic inflammatory signaling (Becker et al., 2015; González-Loyola et al., 2021). However, the effects of lymphedema on immune cells during wound healing have not been as well characterized.

In addition, we observed that lymphatic knockdown and immune cell impairment caused changes in the tissue architecture during healing. We found that the healing tissue of *Foxc2* ^+/−^ mice had collagen fibers that were significantly thinner and more highly aligned than the healed tissue of wildtype mice, with highly aligned collagen fibers indicated increased fibrosis and fibrotic tissue (Xue and Jackson, 2015; Chen et al., 2021). Pro-resolving macrophages, present during the later stages of wound healing, could help minimize fibrosis by promoting apoptosis of myofibroblasts and suppressing levels of inflammation (Krzyszczyk et al., 2018; Hinz and Lagares, 2020; Wynn and Barron, 2010). Lower levels of macrophages were found at both POD14 and POD18 in *Foxc2* ^+/−^ mice, likely resulting in higher levels of myofibroblasts and the development of more fibrotic scar tissue.

Overall, our findings indicate that the lymphatic system affects immune cell recruitment to affect downstream healing and fibrosis. Future studies should further investigate the potentially complex temporal interplay between lymphangiogenesis, immune cell recruitment, and fibrotic collagen formation including myofibroblast populations. Future work should also characterize the affect that *FOXC2* dysregulation has on blood vessel morphology and abundance and whether angiogenesis is impaired. Individuals over 65 years old are more likely to develop cancer and are highly susceptible to developing chronic wounds, which contribute to significant morbidity and mortality. Because we found that *FOXC2*, which is tied to cancer metastasis and lymphatic dysregulation, also impairs wound healing and promotes fibrotic tissue architecture, future work should also investigate the connection between these disease states. With FOXC2 proposed as a potential therapeutic target for cancer metastasis, its pleiomorphic downstream systemic effects should be considered against the increased chance of developing nonhealing or slow-to-heal wounds. Further delineation of the microenvironment, cellular events, and molecular signals during normal and *Foxc2*-associated abnormal wound healing will significantly improve clinical therapies targeting this important marker.

## Data Availability

The raw data supporting the conclusions of this article will be made available by the authors, without undue reservation.
