# Supplementary material for: Cancer and lymphatic marker FOXC2 drives wound healing and fibrotic tissue formation
Source: Front Physiol. 2024 Oct 15;15:1427113. doi: 10.3389/fphys.2024.1427113 (PMC11518795; doi:10.3389/fphys.2024.1427113)
Supplement: Supplementary file 1 [file Image1.pdf]

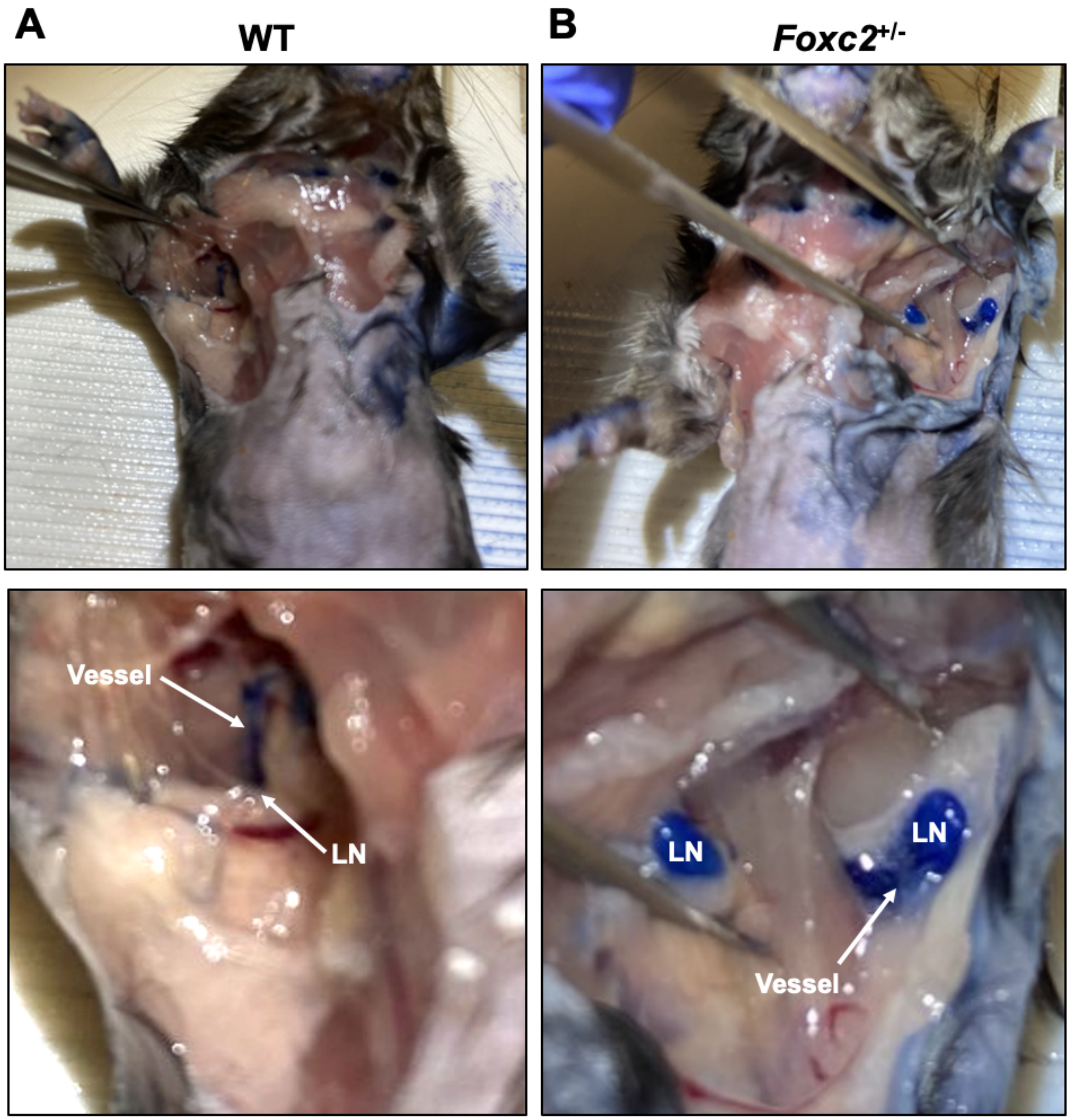

Supplemental Figure 1
Intradermal Evans blue dye (EBD) regional lymphography of **(A)** wildtype and **(B)** *Foxc2*$^{+/-}$ mice.

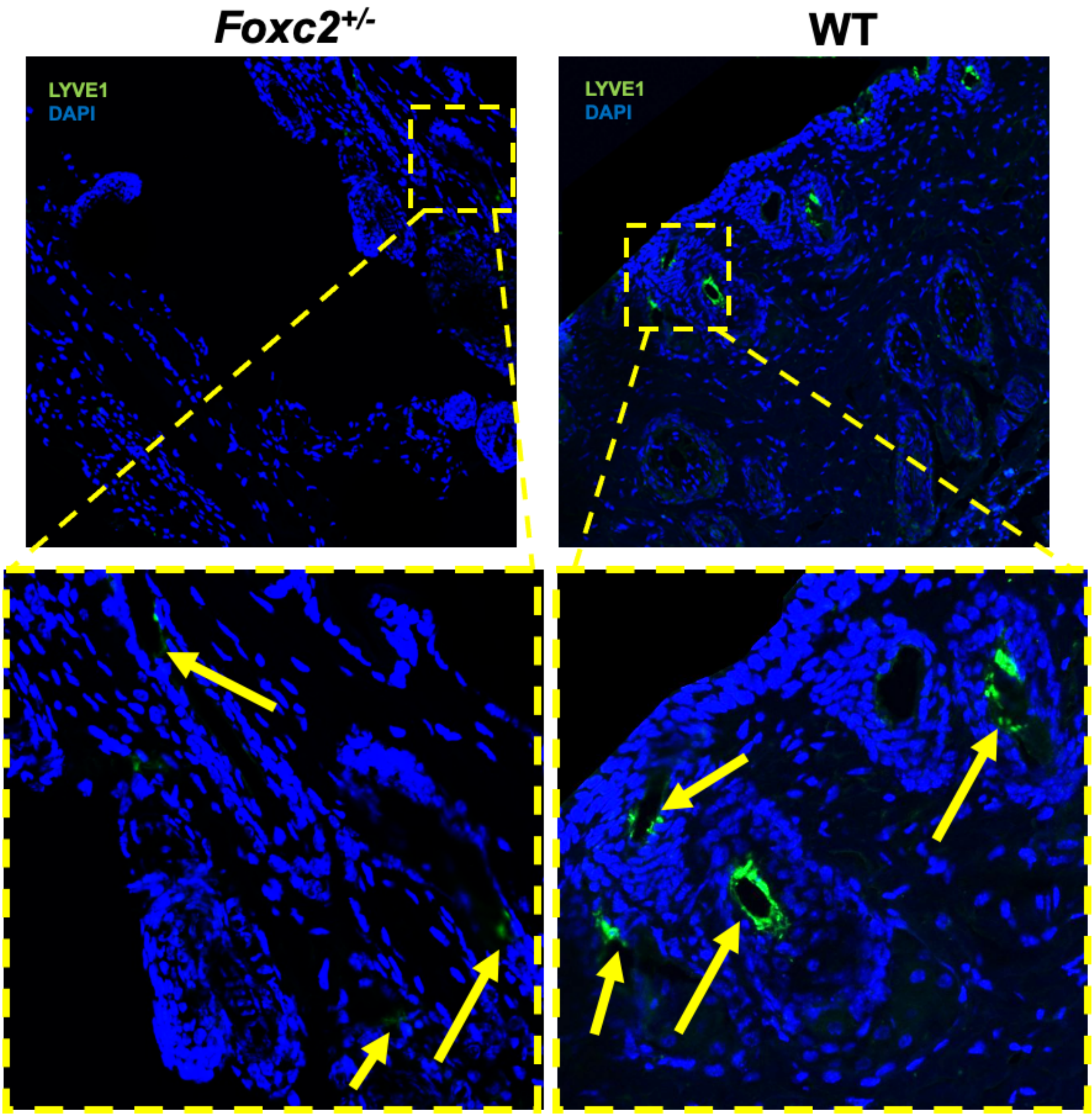

Supplemental Figure 2
Immunostaining for LYVE1 (green) and DAPI (blue) in *Foxc2*$^{+/-}$ and wildtype healed tissue emphasizing lymphatic vasculature.
